# Identification and Characterization of the Most Common Genetic Variant Responsible for Acephalic Spermatozoa Syndrome in Men Originating from North Africa

**DOI:** 10.3390/ijms22042187

**Published:** 2021-02-22

**Authors:** Caroline Cazin, Yasmine Boumerdassi, Guillaume Martinez, Selima Fourati Ben Mustapha, Marjorie Whitfield, Charles Coutton, Nicolas Thierry-Mieg, Pierre Di Pizio, Nathalie Rives, Christophe Arnoult, Aminata Touré, Pierre F. Ray, Raoudha Zouari, Christophe Sifer, Zine-Eddine Kherraf

**Affiliations:** 1 Institute for Advanced Biosciences, INSERM, CNRS, Université Grenoble Alpes, F-38000 Grenoble, France; EXT-CCazin@chu-grenoble.fr (C.C.); gmartinez@chu-grenoble.fr (G.M.); marjorie.whitfield@inserm.fr (M.W.); CCoutton@chu-grenoble.fr (C.C.); christophe.arnoult@univ-grenoble-alpes.fr (C.A.); aminata.toure@inserm.fr (A.T.); PRay@chu-grenoble.fr (P.F.R.); 2 UM GI-DPI, CHU Grenoble Alpes, F-38000 Grenoble, France; 3 Department of Reproductive Biology, Hôpital Jean Verdier, Assistance Publique, Hôpitaux de Paris, F-75004 Paris, France; yasmine.boumerdassi@hotmail.fr (Y.B.); christophe.sifer@jvr.aphp.fr (C.S.); 4 UM de Génétique Chromosomique, CHU Grenoble Alpes, F-38000 Grenoble, France; 5 Centre d’Aide Médicale à la Procréation, Polyclinique les Jasmin, Centre Urbain Nord, Tunis 1003, Tunisia; fourati_selima@yahoo.fr (S.F.B.M.); raouzou@gmail.com (R.Z.); 6 TIMC-IMAG, CNRS, Université Grenoble Alpes, F-38000 Grenoble, France; Nicolas.Thierry-Mieg@imag.fr; 7 EA 4308 ‘Gametogenesis and Gamete Quality, Normandie University, UNIROUEN, F-76000 Rouen, France; pierre.di-pizio@chu-rouen.fr (P.D.P.); Nathalie.Rives@chu-rouen.fr (N.R.); 8 Reproductive Biology Laboratory-CECOS, Assisted Reproductive Center, Rouen Normandy University Hospital, F-76000 Rouen, France

**Keywords:** acephalic spermatozoa syndrome, genetics of male infertility, teratozoospermia, SUN5, whole exome sequencing

## Abstract

Acephalic spermatozoa syndrome (ASS) is a rare but extremely severe type of teratozoospermia, defined by the presence of a majority of headless flagella and a minority of tail-less sperm heads in the ejaculate. Like the other severe monomorphic teratozoospermias, ASS has a strong genetic basis and is most often caused by bi-allelic variants in *SUN5* (Sad1 and UNC84 domain-containing 5). Using whole exome sequencing (WES), we investigated a cohort of nine infertile subjects displaying ASS. These subjects were recruited in three centers located in France and Tunisia, but all originated from North Africa. Sperm from subjects carrying candidate genetic variants were subjected to immunofluorescence analysis and transmission electron microscopy. Moreover, fluorescent in situ hybridization (FISH) was performed on sperm nuclei to assess their chromosomal content. Variant filtering permitted us to identify the same *SUN5* homozygous frameshift variant (c.211+1_211+2dup) in 7/9 individuals (78%). *SUN5* encodes a protein localized on the posterior part of the nuclear envelope that is necessary for the attachment of the tail to the sperm head. Immunofluorescence assays performed on sperm cells from three mutated subjects revealed a total absence of SUN5, thus demonstrating the deleterious impact of the identified variant on protein expression. Transmission electron microscopy showed a conserved flagellar structure and a slightly decondensed chromatin. FISH did not highlight a higher rate of chromosome aneuploidy in spermatozoa from *SUN5* patients compared to controls, indicating that intra-cytoplasmic sperm injection (ICSI) can be proposed for patients carrying the c.211+1_211+2dup variant. These results suggest that the identified *SUN5* variant is the main cause of ASS in the North African population. Consequently, a simple and inexpensive genotyping of the 211+1_211+2dup variant could be beneficial for affected men of North African origin before resorting to more exhaustive genetic analyses.

## 1. Introduction

Infertility is a major public health concern affecting approximately 15% of couples worldwide [1](Mascarenhas et al., 2012). A male factor is implicated in nearly half of all cases and we estimate that over 20 million men in the world are affected by reproductive disorders [2]. Spermatogenic defects resulting in the insufficient production of functional spermatozoa represent a major cause of male infertility. Among these defects, monomorphic or homogeneous teratozoospermia are most likely to be caused by monogenic disorders [3,4].

Acephalic spermatozoa syndrome (ASS; OMIM—Online Mendelian Inheritance in Man: 617187) is a rare but extremely severe type of monomorphic teratozoospermia characterized by the presence of a majority of headless flagella and a minority of tail-less sperm heads in the ejaculate [5]. In addition to familial cases, many knock-out mouse models recapitulate the human phenotype of ASS, demonstrating a monogenic inheritance [6]. In the last few years, the development and availability of high throughput sequencing (HTS) techniques such as whole exome sequencing (WES) has allowed the identification of pathogenic or likely pathogenic variants in several genes: *SUN5, BRDT, PMFBP1, TSGA10* and *HOOK1* [6].

*SUN5* was the first gene reported in ASS, and with over 10 previously reported biallelic variants, remains by far the main cause of ASS (Table S1) [7,8,9,10,11,12].

*SUN5* (Sad1 and UNC84 domain-containing 5), also known as *SPAG4L/TSARG4* (HGNC: 16252; MIM: 613942), is located on chromosome 20q11.21 and its expression is mostly testis-specific [13,14]. The encoded protein is expressed during spermiogenesis and remains present in mature sperm. SUN5 is a transmembrane protein localized to the junction between the sperm nucleus and the head–tail coupling apparatus (HTCA) [15]. As a result, SUN5 deficiency leads to disruption of the sperm head–tail junction and the detachment of the head from the flagellum during spermiogenesis [11,16]. Because most separated sperm heads are retained in the seminiferous epithelium and phagocytized by Sertoli cells, they are rarely observed in the ejaculate.

In the present study, we analysed nine unrelated men originating from North Africa and displaying ASS associated with primary infertility. We identified a very rare but ASS-frequent homozygous frameshift variant of SUN5 in seven subjects (78%). Following the identification of this variant (c.211+1_+2dupGT (p.Ser71Cysfs11*)), we performed additional experiments to demonstrate its pathogenicity and study its impact on sperm morphology, ultrastructure, and ploidy status.

## 2. Results

### 2.1. Identification of a Rare Homozygous SUN5 Variant in Seven ASS Subjects

**Figure 1:**
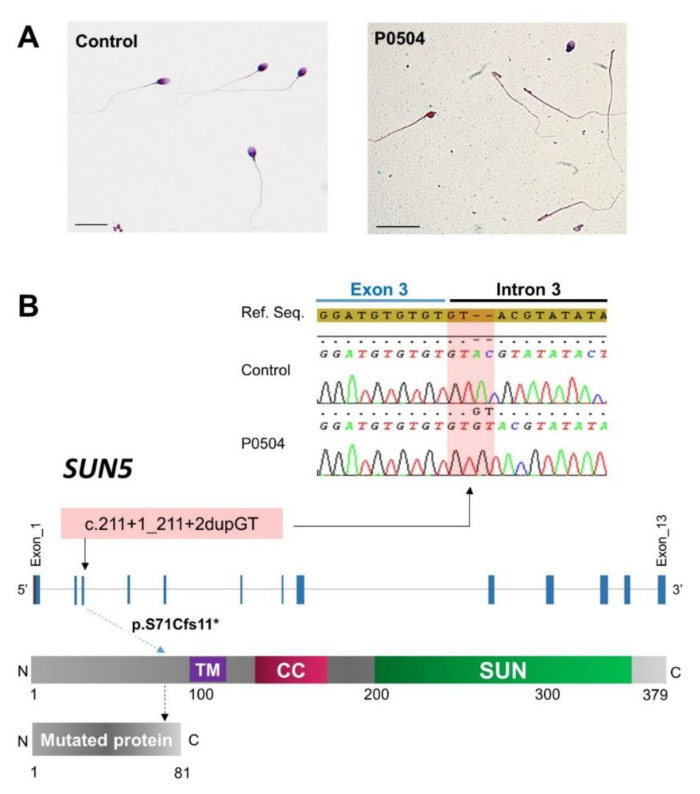
Sperm morphology and a description of the identified *SUN5* variant. (**A**) Light microscopy analysis of spermatozoa from a fertile control individual and a subject carrying the identified *SUN5* variant (P0504). Mutated subjects have numerous isolated flagella and few isolated abnormal heads. Scale bars = 20 µm. (**B**) The identified homozygous variant, as shown in the electropherograms, is located within a GT repetitive sequence located at the junction of exon 3 and the downstream intron (intron 3). We observe that the end of the exon 3 sequence contains three repetitive GT doublets, followed by the consensus donor splice-site sequence (GT) in intron 3. The identified variant causes the extension of this repetitive sequence and is predicted to impact both splicing and translation, leading to the production of a short and non-functional SUN5 protein. The purple box represents a transmembrane domain (106–122 aa). The pink box represents a coiled-coil domain (155–183 aa), and the green box represents the Sad1/UNC-like C-terminal domain (205–364 aa) according to the Ensembl database (protein domains for ENSP00000348496.3). The red box represents the predicted protein in silico.

We recruited nine unrelated infertile men originating from North Africa with an ASS characterized by the presence of a high proportion of headless tails and isolated heads (Figure 1A). Sperm parameters of the nine studied subjects are summarized in Table 1.

Among the nine recruited patients, seven were subjected to WES analysis and Sanger sequencing for validation. Because the ASS phenotype is rare and deleterious variants are expected to be negatively selected, we excluded all variants with a frequency greater than 1% in the gnomAD database. We then considered all the truncating variants as potentially deleterious, all variants scored as deleterious or probably damaging by sorting intolerant from tolerant (SIFT) and/or polymorphism phenotyping (PolyPhen), respectively, and all exonic or intronic near exonic variants were predicted to have a significant impact on splicing (predicted to create or obliterate a donor or acceptor splice site and scored as probably affecting splicing or potentially altering splicing) by HSF (Human Splicing Finder).

Finally, we retained a rare homozygous truncating variant of *SUN5* (ENST00000356173.8; c.211+1_211+2dupGT) in five subjects (P0166, P0168, P0177, P0386, and P0504) which is predicted to shift the reading frame, leading to the production of a truncated and non-functional protein (Figure 1B).

This variant was absent in all subjects of our control cohort (*n* = 485). In addition, it has only been reported in the gnomAD database in the heterozygous state and with an allelic frequency (AF) of 0.0166%. In the two remaining patients, we did not identify any bi-allelic deleterious variant in potential candidate genes, which were defined as follows: (1) other genes involved in acephalic spermatozoa syndrome, particularly in *PMFBP1 and TSGA10* [17,18,19,20]; (2) genes previously described in a context of male infertility; or (3) genes with a predominant testicular expression or restricted expression in the testes.

Because we identified this variant in five subjects (71%), we performed targeted sequencing on the last two subjects (PS1 and PS2). Both were homozygous for the c.211+1_211+2dupGT variant (Figure 1B). Thus, we identified this variant in seven out of nine patients, increasing the diagnosis yield to 78%.

### 2.2. Dating of the SUN5 c.211+1_211+2dupGT Variant

To estimate the age of the *c.211+1_211+2dupGT* variant identified in ASS infertile men, we used the size of the shared ancestral haplotype surrounding the *SUN5* insertion locus. Allowing for a single artefactual variant call (chr20:32943766 G→A in one sample), we observed a shared stretch of homozygosity in all five affected individuals who were analyzed by WES and expressed this variant in the homozygous state. This stretch of homozygosity starts between coordinates 32826214 and 32836863 and finishes between coordinates 33008666 and 33015599. We therefore estimate the shared haplotype size at approximately 181 Kb, which corresponds to 0.24 cM assuming a recombination rate of 1.30 cM/Mb [21].

The probability of observing a single ancestral haplotype of size *c* (measured in morgans) or larger is equal to (1−*c*)g, where *g* is the age of the deletion, measured in generations [22]. Thus, the probability that the 2*n* haplotypes carried by *n* unrelated individuals is larger than *c* is (1−*c*)2ng, which can be approximated by *e*−2ngc. Therefore, the length of the shared ancestral haplotype can be approximated by an exponential distribution of rate 2*n*g**, leading to g ≈ 1/(2*nc*). Here, we have *n* = 5 individuals and *c* = 0.0024 M*,* which provides an estimate of *g* = 42 generations for the *SUN5* variant. Labuda et al. have shown that dating arising from genetic clock methods is biased downward because these methods do not account for population expansion [23]. To account for an exponential expansion of rate ***r*** per generation, the mathematical expression (−1/***r***)ln(***c***.***e***r/(***e***r − 1)) should be added to the estimates provided by genetic clock methods. If we assume that an upper bound for the expansion rate is given by the expansion ***r*** = 0.4, as was calculated for the Ashkenazi Jewish population [24], the age of the mutation is in the range of 42–54 generations. Given 25 years for the generation time, the mutation would have then arisen 1050–1350 years ago.

### 2.3. Demonstration of the Pathogenicity of the c.211+1_211+2dupGT SUN5 Variant

**Figure 2:**
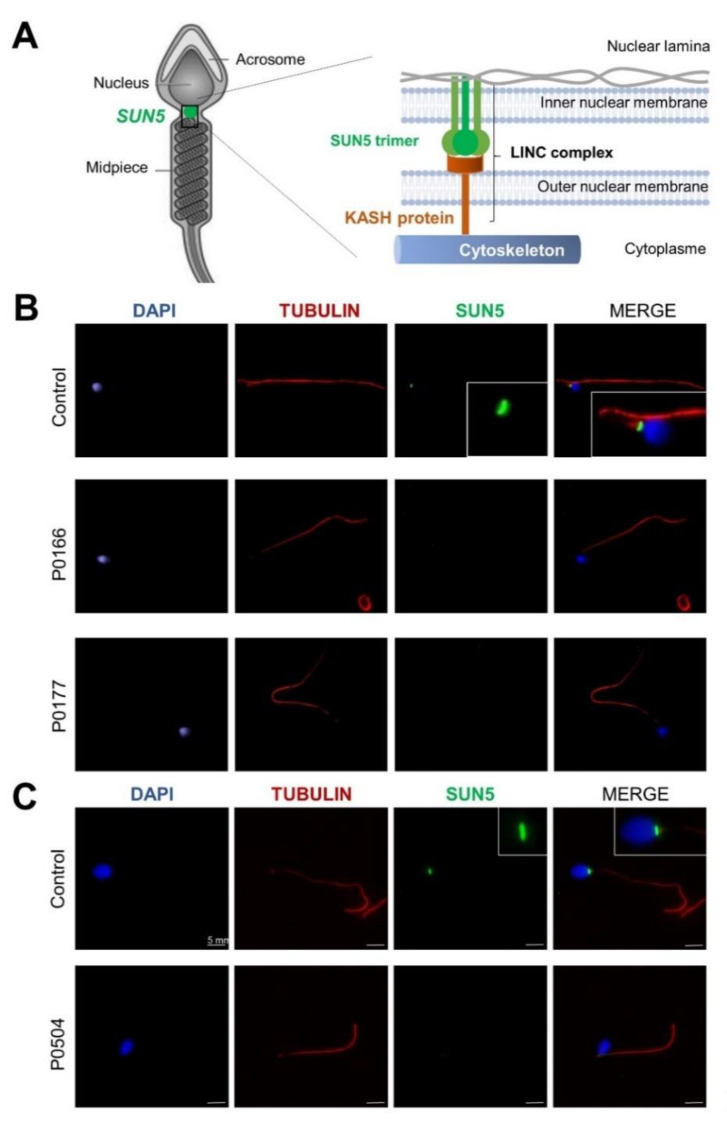
Localization of SUN5 and immunostaining in human spermatozoa from control and mutated subjects. (**A**) Localization of the SUN5 protein at the base of the sperm head, where it functions to anchor the sperm head to the flagellum. (**B**,**C**) Sperm cells from mutated subjects P0166 and P0177 (**B**) and P0504 (**C**) in addition to those from fertile controls were stained with anti-SUN5 (1:200, green) and anti-acetylated tubulin (1:2000, red) antibodies. DNA was counterstained with 4′,6-diamidino-2-phenylindole (DAPI), showing sperm nuclear DNA (blue). The SUN5 immunostaining (green) marked a punctiform signal at the head–tail junction. The co-staining with alpha-tubulin, specific for the flagella, confirm the localization. In contrast, the SUN5 signal is absent from P0166, P0177, and P0504, confirming the deleterious effect of the mutation. KASH: Klarsicht, ANC-1, Syne Homology; LINC: linker of nucleoskeleton and cytoskeleton.

The SUN5 protein was shown to localize to the inner nuclear membrane and to interact with protein containing Klarsicht, ANC-1, Syne Homology (KASH) domains to form linker of nucleoskeleton and cytoskeleton (LINC) complexes between the outer and the inner nuclear membranes (Figure 2A). In order to assess the deleterious impact of the identified *SUN5* variant and its implication in ASS and male infertility, we used immunofluorescence staining to study the localization and the level of SUN5 protein expression in sperm cells from three mutated subjects (P0386, P0166 and P0504) compared to controls. As expected, in controls, SUN5 staining yielded a punctiform signal at the head–tail junction (Figure 2B,C). The co-staining of β-acetylated tubulin, specifically marking the flagella, confirms the localization. In contrast, in sperm from mutated subjects, we did not observe any SUN5 signaling, confirming the deleterious effect of the mutation.

### 2.4. Ultrastructural Sperm Defects Associated with the Identified Homozygous SUN5 Variant

**Figure 3:**
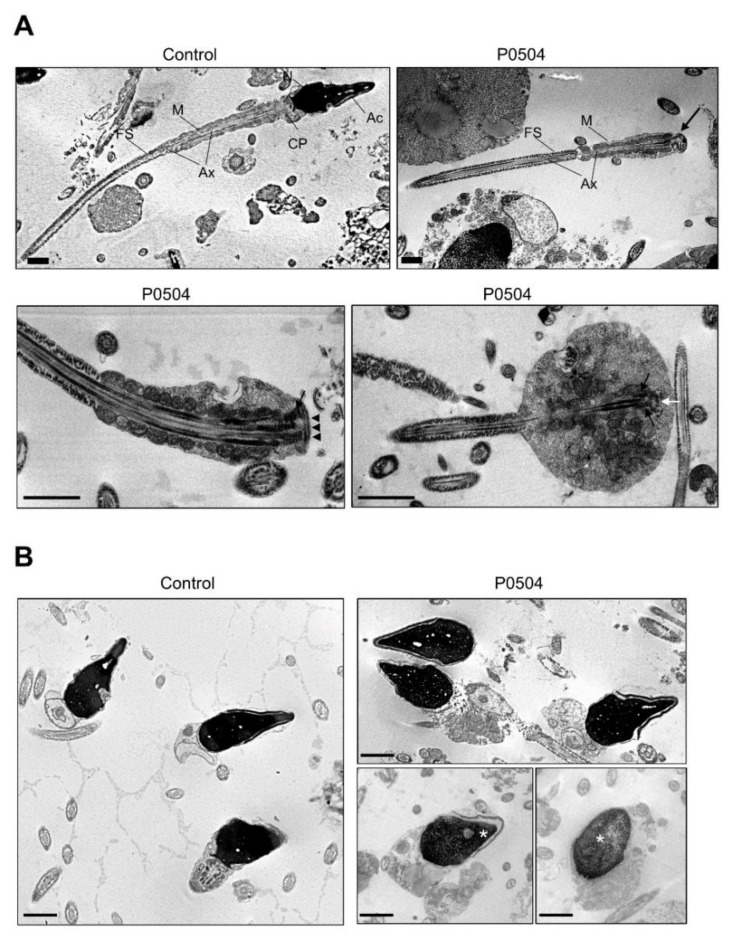
Sperm ultrastructure analysis by transmission electron microscopy (TEM). TEM analysis of semen samples from a control individual and from individual P0504. (**A**) Longitudinal sections of sperm flagella showing an overall regular organization of the midpiece and principal piece in the sample from individual P0504, although some flagella were identified with cytoplasmic remnants and an irregular mitochondrial sheath. The head–tail junction was also clearly distinguishable, as were the centriole (white arrow) and the striated columns (black arrow); the basal plate was also visible (black arrowheads). Ac: acrosome; N: nucleus; MS: mitochondrial sheath; Ax: axoneme; FS: fibrous sheath. Scale bars represent 1 µm. (**B**) Analysis of isolated heads in the semen sample from individual P0504 showing normal acrosomal structure but altered chromatin condensation (white asterisk). Scale bars represent 1 μm.

To study the ultra-structure of sperm from *SUN5* mutated ASS individuals, we performed transmission electron microscopy (TEM) analysis on a fresh ejaculated semen sample from individual P0504. We first observed that despite head detachment, isolated sperm flagella from ASS individuals appeared globally normal and harbor distinctive midpiece and principal piece compartments, similar to that observed in sperm from control individuals. As observed with optic microscopy and previously described by others [5], a remnant of cytoplasm was sometimes present, and associated with an irregular mitochondrial sheath (Figure 3A). When looking more closely at the head–tail junction, the centriole and pericentriolar structures known as the striated columns could sometimes be distinguished; the basal plate was also visible (Figure 3A). The quantification of transversal sections of the axoneme indicated that 72% of sections had a normal microtubule conformation (9 + 2) as compared to an average of 84.5% for control individuals (*n* = 4; range: 76–92). Importantly, isolated heads had a normal acrosomal structure, but chromatin condensation appeared to be altered more often (Figure 3B). Quantification of the semen sample from individual P0504 indicated that 38% of sperm heads had normal chromatin condensation, as compared to an average of 65% for control sperm (*n* = 4; range: 46–82).

### 2.5. FISH Did Not Demonstrate a Higher Rate of Sperm Chromosomal Aneuploidies in ASS Men Carrying the Identified SUN5 Variant

**Figure 4:**
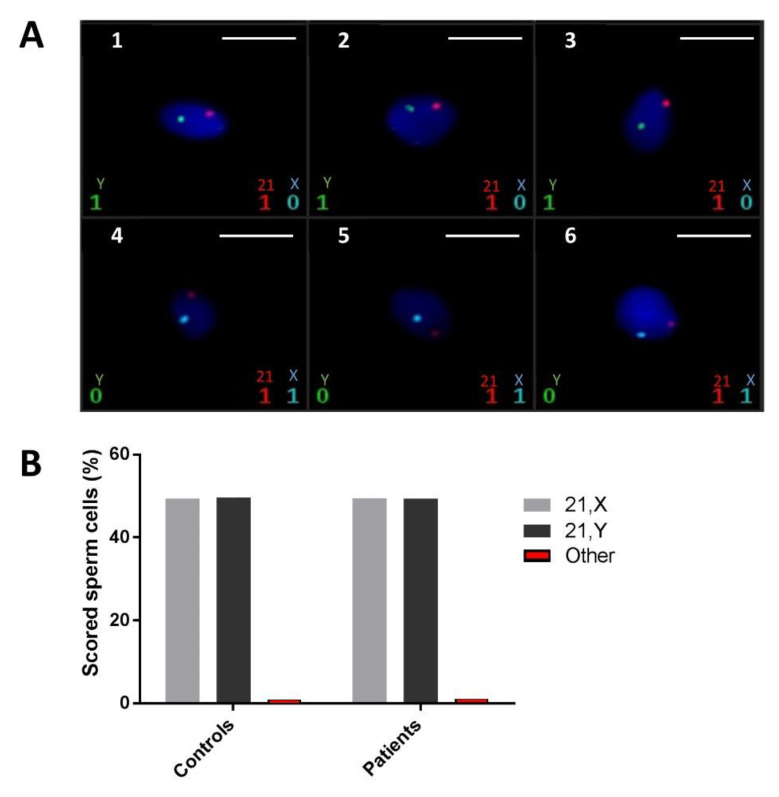
Sperm fluorescent in situ hybridization (FISH). (**A**) Images 1–6 show normal X, Y (blue and green spots, respectively) bearing sperm with no aneuploidy. Chromosome 21 is present every time (in red). Scale bars represent 5 µm. (**B**) Percentages of normal cells (21,X or 21,Y) and percentages of other types of ploidy (disomic for sex chromosomes, chromosome 21, or diploid cells).

FISH analysis of ejaculated spermatozoa was carried out using three different probe sets for chromosomes X, Y, and 21 in various colors. X and Y chromosomes are respectively represented by blue and green spots; the red spots represent chromosome 21. Sperm chromatin was stained with 4′,6-diamidino-2-phenylindole (DAPI) and fluoresces in dark blue (Figure 4A). The analysis was carried out on a total of 24,866 spermatozoa obtained from five fertile controls and 6355 spermatozoa from three ASS subjects carrying the identified *SUN5* variant. At least 2000 cells were counted per sample, with a mean of 3831 cells. As hybridization defects cannot be differentiated from nullosomic cells, only disomic or diploid cells were considered. Each sperm cell was identified as normal (21,X or 21,Y), disomic for sex chromosomes (21,X,Y or 21,X,X or 21,Y,Y), disomic for chromosome 21 (21,21,X or 21,21,Y) or diploid (21,21,X,Y or 21,21,X,X or 21,21,Y,Y) (Table S3). Data analysis did not demonstrate a significant statistical difference between the two groups regarding the aneuploidy rate, thus indicating that chromosomal content, here assessed by chromosomes X, Y, and 21, is not affected in sperm cells produced by men displaying ASS due to SUN5 deficiency.

## 3. Discussion

In the present study, we investigated a cohort of nine infertile men, all originating from North Africa and displaying ASS, by WES and targeted sequencing. This strategy allowed us to identify a frequent homozygous loss-of-function variant of *SUN5* in seven out of nine (78%) analyzed subjects. This result strongly confirms that *SUN5* is the main genetic actor of ASS and that the identified causative variant is responsible for the majority of ASS cases in the North African population. By searching the literature, we found that the same variant was previously identified in a single Chinese case of ASS [8]. However, the deleterious impact of this variant was not validated by functional assays and was just reported with other *SUN5* candidate variants in this previously published study.

**Figure 5:**
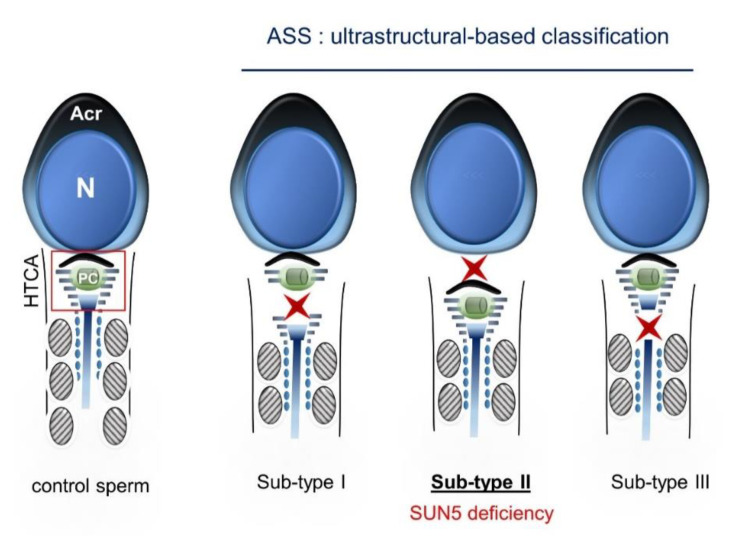
Ultrastructural-based classification of acephalic spermatozoa syndrome (ASS). Red star markers indicate the breakage level of the head–tail coupling apparatus (HTCA), defining three ASS sub-types. SUN5 deficiency leads to sub-type II, marked by the disruption of the junction between the nuclear envelope and the proximal centriole (PC). Acr: acrosome; N: nucleus.

In sperm from control subjects, immunofluorescence staining showed a punctiform signal, thus confirming the localization of SUN5 at the head–tail junction. As expected, the signal was absent in sperm from ASS individuals carrying the *SUN5* variant. This absence of signal confirms the deleterious impact of the identified variant. Two hypotheses can explain the observed effect: (1) the duplication of the donor splice site introduces two additional nucleotides in the mRNA sequence, leading to a frameshift and the production of a truncated protein (p.S71Cfs11*). The truncated protein would therefore be lacking its SUN domain (from 205–364 aa) and could not localize to the nuclear membrane; or (2) the variant leads to the production of unstable mRNA that is degraded by nonsense-mediated mRNA decay (NMD). Unfortunately, as the studied patients had only a few sperm heads, we could not extract enough mRNA to perform a successful RT-PCR and determine which of these hypotheses is correct. Finally, to demonstrate the deleterious impact of this homozygous variant, we performed a series of immunofluorescence (IF) assays using a highly sensitive and specific antibody raised against SUN5 (17495–1-AP; Proteintech) that has been previously validated and successfully used in several published studies [8,12,14,15,16,17,20]. SUN5 is a member of the SUN domain-containing protein family, which comprises five members (SUN1–5). These proteins are transmembrane proteins that localize at the inner nuclear membrane and directly interact with KASH domain proteins to form LINC complexes between the outer and inner nuclear membranes in order to connect the nucleus to the cell cytoskeleton [25]. SUN5 is highly and specifically expressed in the testes and shows an increased transcriptional expression level during the last step of germ cell development, concomitant with the round spermatid stage [13,15]. The encoded protein localizes specifically at the posterior nuclear region toward the head–tail junction, a structure termed the head–tail coupling apparatus (HTCA) [15]. The HTCA originates from the centrosome and connects the nuclear envelope to the intermediate piece of the flagellum [26]. SUN5 deficiency is associated with a normal assembly of the HTCA during the early stages of spermiogenesis, but it dissociates from the nuclear envelope in elongated spermatids, causing the detachment of the tail from the head [27]. The ultrastructural analysis of ejaculated spermatozoa from subject P0504 carrying the identified variant also showed that the HTCA was normally assembled but completely detached from the sperm nuclear envelope. Based on the ultrastructure of acephalic spermatozoa observed in several ASS cases, this phenotype could be classified into three sub-types: sub-type 1) acephalic spermatozoa with decapitated heads that contain only the proximal centriole; sub-type 2) acephalic spermatozoa with decapitated tails that contain the proximal and distal centriole; and sub-type 3) acephalic spermatozoa with decapitated heads that contain the proximal and distal centrioles (Figure 5).

**Figure 6:**
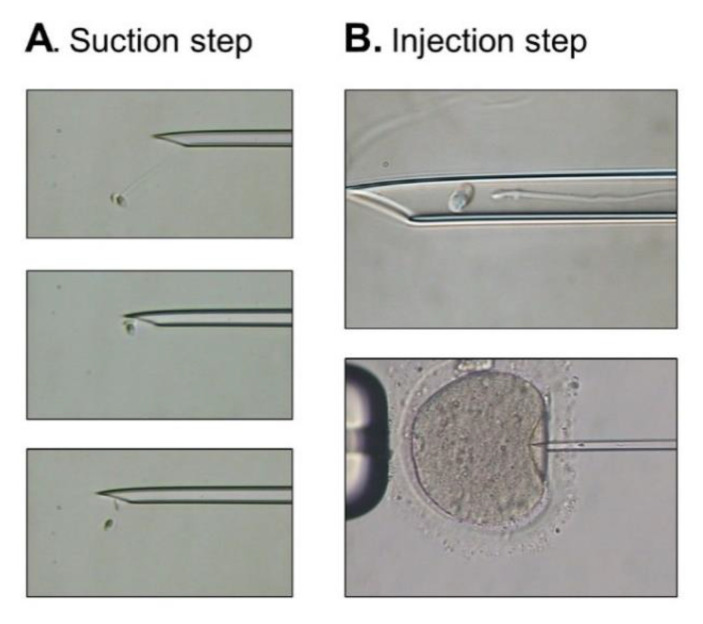
Intracytoplasmic sperm injection (ICSI) procedure using selected sperm from subject P0504. (**A**) A complete spermatozoon showing progressive motility was selected. Gentle immobilization was performed, and suction to aspirate the spermatozoon into the injection needle was first applied to the tail. As the sperm head sheared from the tail during suction, the next step consisted of suctioning the head to place it with the tail in the injection needle. (**B**) Both the head and the tail were injected within the same needle into a mature oocyte.

The ultrastructure of acephalic spermatozoa from individual P0504 observed by TEM clearly demonstrates ASS sub-type II, which is concordant with SUN5 deficiency. In this case, isolated sperm heads observed in the ejaculate are lacking the centrioles. The importance of these structures in human reproduction is still debated, but it seems important for successful fertilization and embryonic development [28]. Some reports indicate successful In vitro fertilization with intracytoplasmic sperm injection (IVF-ICSI) for ASS patients performed with isolated heads, while others indicate the use of a fully assembled spermatozoa or the addition of both an isolated head and an isolated flagellum, as performed for patient P0504 (Figure 6). Indeed, for this patient, two ICSI cycles retrieving 17 oocytes in all and 14 metaphase two-stage oocytes led to 100% fertilization and cleavage rates associated with the delivery of three healthy babies. Further quantitative analyses would have to be performed to determine if the co-injection of the head and the flagellum is necessary during ICSI for ASS subjects.

To complete our phenotypic characterization of sperm produced by subjects carrying the identified *SUN5* variant, we analyzed the chromatin content of ejaculated spermatozoa from three mutated subjects (P0166, P0386 and PS1) by fluorescent in situ hybridization (FISH). Results showed that sperm from these subjects had a low rate of aneuploidy, which is comparable with that observed in the sperm of fertile controls (*n* = 5). This result is also in favor of good IVF-ICSI outcomes for ASS subjects.

In conclusion, our results confirm that *SUN5* is the main causal gene of ASS and that WES is efficient to identify single gene defects in a context of severe male infertility. Moreover, we report here a frequent *SUN5* variant that could be responsible for nearly 80% of ASS cases in the North African population. The effect of this variant cannot be questioned as: (1) it was identified in seven unrelated individuals; (2) it affects a consensus splice donor site and is predicted to introduce a frameshift in the mRNA sequence; (3) the protein was shown to be absent in mature sperm from mutated subjects; and (4) it was previously identified through an independent study in an infertile man displaying ASS and originating from a distant geographical region (China), thus definitively confirming its causative role in ASS and primary male infertility.

## 4. Material and Methods

### 4.1. Subject Selection and Recruitment

Nine unrelated infertile men were included in the present study, all originating from North Africa and displaying non-syndromic infertility due to acephalic spermatozoa syndrome (ASS). Subjects were recruited from three reproductive biology centers: seven individuals sought consultation for primary infertility at the Clinique des Jasmins in Tunis (Tunisia), one individual was recruited at the Jean Verdier Hospital in Paris (France), and one individual was recruited at the Rouen University Hospital (France).

Informed consent was obtained from all individuals participating in the study according to local protocols and the principles of the Declaration of Helsinki. The study was approved by local ethics committees, and samples were then stored in the Centre de Resources Biologiques Germethèque (certification under ISO-9001 and NF-S 96–900) according to a standardized procedure, or were part of the Fertithèque collection declared to the French Ministry of Health (DC-2015–2580) and the French Data Protection Authority (DR-2016–392).

### 4.2. Semen Parameters and Sperm Morphology

Semen samples were obtained by masturbation after two to seven days of sexual abstinence. Semen samples were incubated for 30 min at 37 °C for liquefaction; ejaculate volume and pH, together with sperm concentration, vitality, morphology, and motility, were evaluated according to World Health Organization (WHO) guidelines [29]. Sperm vitality was assessed by eosin staining, and sperm morphology was analyzed on Schorr stained semen smears according to David’s modified classification [30].

### 4.3. Whole Exome Sequencing and Variant Filtering

Genomic DNA was isolated from blood samples or saliva using the Oragen DNA Extraction Kit (DNA Genotek Inc., Ottawa, ON, Canada). Genetic data were obtained from various sequencing centers, in particular Novogene and Integragen. Coding regions and intron/exon boundaries were sequenced after enrichment using SureSelect Human All Exon V6 from Agilent.

An alignment-ready GRCh38 reference genome (including alternative sequences (ALT), decoy, and human leukocyte antigens (HLA)) was produced using “run-gen-ref hs38DH” from Heng Li’s bwakit package [31]. The exomes were analyzed using a bioinformatics pipeline developed in-house. The pipeline consists of two modules, both distributed under the GNU General Public License v3.0 and available on github.

The first module (https://github.com/ntm/grexome-TIMC-Primary (accessed on 15 January 2021)) takes FASTQ files as input and produces a single merged GVCF file, as follows. Adaptors are trimmed and low-quality reads are filtered with fastp 0.20.0 [32], reads are aligned with BWA-MEM 0.7.17 [33], duplicates are marked using samblaster 0.1.24 [34], and BAM files are sorted and indexed with samtools 1.9 [35]. SNVs (single nucleotide variants) and short indels are called from each BAM file using strelka 2.9.10 to produce individual GVCF files [36]. These are finally merged with mergeGVCFs.pl to obtain a single multi-sample GVCF, which combines all exomes available in our laboratory.

The second module (https://github.com/ntm/grexome-TIMC-Secondary (accessed on 15 January 2021)) takes this merged GVCF as input and produces annotated analysis ready TSV files. This is achieved by performing up to 15 streamlined tasks, including the following: low-quality variant calls (read depth (DP) < 10, genotype quality for variant filtration (GQX) < 20, or less than 15% of reads supporting the ALT allele) are discarded; Variant Effect Predictor v92 (McLaren et al., 2016) is used to annotate the variants and predict their impact, allowing us to filter low-impact variants and/or prioritize high-impact ones (e.g., stop-gain or frameshift variants) [37]; gene expression data from the Genotype-Tissue Expression project (GTEx v7) are added; variants with a minor allele frequency greater than 1% in gnomAD v2.0, 3% in 1000 Genomes Project phase 3, or 5% in NHLBI (National Heart, Lung, and Blood Institute, Bethesda, MD, USA) ESP6500 are filtered (variants are also compared to those obtained from 485 exomes of healthy control individuals or of patients presenting a clearly different phenotype. Because all variants result from the same bioinformatics pipeline, this allows us to filter artifacts due to the pipeline itself, as well as genuine variants that may be missing from public databases but are actually not so rare in our cohorts); finally, the resulting TSV files can be opened with spreadsheet software such as LibreOffice Calc or Microsoft Excel for further filtering and sorting, in order to identify candidate causal variants.

### 4.4. Sanger Verification of the Variant

Variants identified by WES were subjected to Sanger verification using an ABI 3500XL Genetic Analyzer (Thermo Fisher Scientific, Waltham, MA, USA). Analyses were performed using SeqScape software 3.0 (Applied Biosystems, Foster City, CA, USA). Primer sequences and their expected product sizes are summarized in Table S2.

### 4.5. Immunofluorescence Analysis of Sperm Cells

Immunostaining was carried out on samples from individuals P0386, P0166, and P0504. Sperm cells were fixed in phosphate buffered saline (PBS) and 4% paraformaldehyde (PFA) for 7 min. After washes in PBS, the sperm suspension was spotted onto slides pre-coated with 0.1% poly-l-lysine (Thermo Fisher Scientific). Heat antigen retrieval was performed by boiling slides immersed in 0.01 M sodium citrate buffer, 0.05% Tween 20, pH 6.0 for 20 min. Slides were then blocked in 5% normal goat serum (NGS; GIBCO, Thermo Fisher Scientific) + DPBS for 1 h at room temperature. Incubation with primary antibodies was performed overnight at 4 °C in 0.1% (*v*/*v*) Triton X-100 + DPBS (Triton X-100, MilliporeSigma, Ontario, Canada) with 2% NGS. Polyclonal rabbit anti-SUN5 antibody was purchased from Proteintech, Manchaster, UK, (17495–1-AP) and diluted at 1:200. Monoclonal mouse anti-acetylated α-tubulin antibody was purchased from Sigma-Aldrich (Irvine, UK) and diluted at 1:2000. The slides were then washed and incubated with secondary antibody diluted at 1:800 (Dylight-549-conjugated goat anti-mouse immunoglobulin G (IgG) and Dylight-488-conjugated goat anti-rabbit IgG, Jackson ImmunoResearch (Cambridgeshire, United Kingdom) and Hoechst 33342 (Sigma-Aldrich) for 1 h at room temperature, rinsed, and mounted with Dako Fluorescence Mounting Medium (Thermo Fisher Scientific). Images were acquired on a Nikon Eclipse 8 microscope and processed with ISIS software.

### 4.6. Transmission Electron Microscopy

To better characterize the impact of the reported candidate variant on sperm ultrastructure, sperm from P0504 (an individual carrying this variant) were subjected to transmission electron microscopy. Sperm were collected from fresh ejaculate and washed with M2 medium (Sigma-Aldrich Co. Ltd., Irvine, UK) by centrifugation at 300 g for 10 min. Sperm cells were fixed in 0.1 M phosphate buffer, pH 7, containing 3% glutaraldehyde (Grade I; Sigma-Aldrich Co., St. Louis, MO, USA) (room temperature, 90 min). After two washes in PBS + 1% sucrose, samples were resuspended in 0.2 M sodium cacodylate buffer. Secondary fixation was performed with 1% osmium tetra-oxide (Agar Scientific Ltd., Essex, UK) and was followed by dehydration in graded alcohol and embedding in Epon resin (Polysciences Inc., Warrington, PA, USA). After staining with toluidine blue-Azur II, semi-thin sections were examined on a Zeiss Axioscope photomicroscope (Carl Zeiss, GmbH, Jena, Germany). The use of a Reichert OmU2 ultramicrotome (Reichert-Jung AG, Wien, Austria) allowed us to obtain ultra-thin sections (90 nm), which were then stained with uranyl acetate and lead citrate. These sections were examined with a JEOL JEM 100CX II electron microscope (Jeol Ltd., Tokyo, Japan). All images were acquired with Digital Micrograph software coupled to a Gatan Erlangshen CCD (charge-coupled device) camera.

### 4.7. Sperm Fluorescent In Situ Hybridization (FISH)

FISH was performed on ejaculated spermatozoa from patients P0166, P0386, and PS1, in addition to fertile controls (*n* = 5). Semen samples were prepared for a triple-color FISH using centromeric probes for chromosomes 21, X, and Y (XA TriScore Aneusomy Probe- MetaSystems^®^ Probes, Heidelberg, Germany). Sperm samples were washed in phosphate buffered saline solution and fixed in methanol/acetic acid solution (3:1). Sperm head decondensation was performed using a solution of NaOH (1 M) and then washed twice in saline sodium citrate (SSC) solution. Denaturation was performed on sperm nuclei and probes for 2 min at 75 °C. After overnight incubation at 37 °C, the slides were washed in 0.4× SSC/0.3% NP40, then twice in SSC/0.1% NP40. The slides were counterstained with 4,6-diamino-2-phenylindole (DAPI) and analyzed with a Leica DM 5000B epifluorescence microscope equipped with a Metafer Slide Scanning System and MetaCyte software (Metasystems^®^, Altlussheim, Germany). Chromosomal aneuploidies were analyzed simultaneously for chromosomes 21, X, and Y with Aqua, FITC, and Spectrum Orange filters. All the combinations of spots counted in the cells during the automatic procedure were manually verified by a trained technician in the galleries of images provided by the machine. Statistical analyses were calculated using Student’s t test on GraphPAD prism software 6 (San Diego, CA USA).

## Figures and Tables

**Table 1:** Detailed semen parameters of subjects carrying the *SUN5* homozygous variant (c.211+1_211+2dupGT) and statistical comparison of the mean of the semen parameters between these subjects and those with unknown cause.

Subject Parameters	Lower Reference Limits (WHO, 2010)	P0166	P0168	P0177	P0386	P0504	PS1	PS2	All SUN5 Mutated Subjects (*n* = 7)	All Non-Mutated Subjects (*n* = 2)	p Value
Age	-	37	52	37	42	31	49	38	40.86 ± 7.38 (*n*’ = 7)	39 ± 2.82 (*n*’ = 2)	0.61
Sperm volume (mL)	1.5	1.4	3	7	2.7	3.6	2	2.2	3.12 ± 1.85 (*n*’ = 7)	5.23 ± 2.09 (*n*’ = 2)	0.36
Sperm concentration (million/mL)	15	24	27	13.2	25.6	12.2	6.2	18	17.85 ± 8.16 (*n*’ = 7)	9.76 ± 13.07 (*n*’ = 2)	0.54
Total motility 1 h (%)	40	10	30	30	40	29	40	50	34.86 ± 12.65 (*n*’ = 7)	15 ± 14.14 (*n*’ = 2)	0.26
Vitality (%)	58	83	70	80	65	47	60	80	69.26 ± 13.09 (*n*’ = 7)	51.25 ± 1.77 (*n*’ = 2)	0.29
Typical forms (%)	4	0	0	1	0	0	0	0	0.14 ± 0.38 (*n*’ = 7)	11.25 ± 15.91 (*n*’ = 2)	0.50
Abnormal head (%)	-	84	90	70	84	54	72	74	75.39 ± 12.05 (*n*’ = 7)	51 ± 43.84 (*n*’ = 2)	0.57
Absent flagella (%)	-	48	30	33	52	33	52	46	42.04 ± 9.60 (*n*’ = 7)	21.75 ± 28.64 (*n*’ = 2)	0.50
Bent flagella (%)	-	34	62	52	22	25	40	48	40.35 ± 14.67 (*n*’ = 7)	31.5 ± 4.95 (*n*’ = 2)	0.23
Multiple anomalies index	-	2.8	3.5	2.2	2.7	2.4	2.1	-	2.62 ± 0.51 (*n*’ = 6)	2 ± 2.1 (*n*’ = 2)	0.29

WHO = World Health Organization; SD = standard deviation; *n* = total number of patients in each group; *n*’ = number of patients used to calculate the average based on available data. A significant difference at *p* < 0.05.

## Data Availability

Not applicable.

## Supplementary Materials

The following are available online at https://www.mdpi.com/1422-0067/22/4/2187/s1.
