# Supplementary material for: Identification and Characterization of the Most Common Genetic Variant Responsible for Acephalic Spermatozoa Syndrome in Men Originating from North Africa
Source: Int J Mol Sci. 2021 Feb 22;22(4):2187. doi: 10.3390/ijms22042187 (PMC7927044; doi:10.3390/ijms22042187)
Supplement: Supplementary file 1 [file ijms-22-02187-s001.pdf]

SUPPLEMENTARY TABLES

Table 1. List of mutations previously identified in SUN5 implicated in Acephalic Spermatozoa syndrome.

Gene Name	Chromosome Position	Coding Sequence Variant	Protein Variant	Cohort Description	Mutated Subjects	Reference
**SUN5**	20:31571580-31592240	c.824C>T	p.Thr275Met	17 unrelated men	1	(Zhu et al., 2016)
		c.851C>G	p.Ser284*		1	
		c.340G>A	p.Gly114Arg		1	
		c.1066C>T/ c.485T>A	p.Arg356Cys and p.Met162Lys		1	
		c.381delA/ c.824C>T	p.Val128Serfs*7 and p.Thr275Met		1	
		c.381delA/ c.781G>A	p.Val128Serfs*7 and p.Val261Met		1	
		c.216G>A/ c.1043A>T	p.Trp72* and p.Asn348Ile		1	
		c.425+1G>A/c.1043A>T	p.Asn348Ile		1	
		GRCh38 - chr20:32995761_32990672delinsTGGT	p.Leu143Serfs*30	3 related men	3	(Elkhatib et al., 2017)
		c.829C>T	p.Gln277*	3 unrelated men	1	(Fang et al., 2018)
		c.1067G>A	p.Arg356His		1	
		c.211+1_211+dupGT	p.Ser71Cysfs11*		1	
		c.381delA	p.Val128Serfs*7	15 unrelated men	5	(Sha et al., 2018a)
		c.781G>A/ c.1043A>T	p.Val261Met and p.Asn348Ile	3 unrelated men	1	(Shang et al., 2018)

	c.425+1G>A/c.475C>T	p.Arg159*		1	
	c.772C>T	p.Arg258Cys	11 unrelated men	1	(Liu et al., 2020)

Table S2. Sequences of primers used and expected product sizes

Primer sequences used for Sanger sequencing verification of *SUN5* mutations and respective melting temperatures (Tm) and product sizes.

Primer Name	Primer Sequence (5'-3')	Tm (°C)	Product Size (bp)
SUN5_Ex3-Forward	GTCCGCAACAATGACCAAGC	60	406
SUN5_Ex3-Reverse	AAGAGGGATGACTGGGTGGA	60	

Table S3. Sperm-FISH analysis.

Data were recorded from 24866 sperm cells from fertile controls (*n* = 5) and 6355 from *SUN5* mutated subjects affected by ASS.

	Chrom.	CTRL 1	CTRL 2	CTRL 3	CTRL 4	CTRL 5	SUN5 P0166	SUN5 P0386	SUN5 PS1	Mean CTRL ± SD	Mean SUN5 ± SD	*p*-Value
**% Haploid Sperm**	**21,X**	48,77	49,80	49,26	49,07	50,12	48,57	51,46	48,38	49,41±0,55	49,47±1,73	0,95
	**21,Y**	50,27	49,42	49,69	50,00	49,01	50,30	47,56	50,46	49,68±0,49	49,44±1,63	0,82
**% Disomic Sperm**	**21,XY**	0,46	0,42	0,59	0,60	0,69	0,69	0,67	0,63	0,55±0,11	0,66±0,03	0,08
	**21,XX**	0,08	0,06	0,08	0,10	0,02	0,10	0,00	0,10	0,07±0,03	0,07±0,06	0,09
	**21,YY**	0,12	0,08	0,10	0,10	0,02	0,10	0,04	0,10	0,08±0,04	0,08±0,03	0,86
	**21,21,X**	0,04	0,06	0,04	0,04	0,02	0,05	0,04	0,05	0,04±0.01	0,05±0.00	0,33
	**21,21,Y**	0,08	0,06	0,06	0,04	0,04	0,05	0,09	0,05	0,06±0.02	0,06±0.02	0,72
**% Diploid Sperm**	**21,21,XY**	0,06	0,04	0,08	0,00	0,06	0,10	0,09	0,14	0,05±0.03	0,11±0.03	0,04
	**21,21,XX**	0,08	0,04	0,04	0,02	0,02	0,05	0,00	0,05	0,04±0.02	0,03±0.03	0,71
	**21,21,YY**	0,04	0,02	0,06	0,02	0,00	0,00	0,04	0,05	0,03±0.02	0,03±0.03	0,89
